# Desolvation of the substrate-binding protein TauA dictates ligand specificity for the alkanesulfonate ABC importer TauABC

**DOI:** 10.1042/BCJ20190779

**Published:** 2019-12-10

**Authors:** Feng Qu, Kamel ElOmari, Armin Wagner, Alfonso De Simone, Konstantinos Beis

**Affiliations:** 1 Department of Life Sciences, Imperial College London, South Kensington, London SW7 2AZ, U.K.; 2 Rutherford Appleton Laboratory, Research Complex at Harwell, Oxfordshire OX11 0DE, U.K.; 3 Diamond Light Source, Harwell Science and Innovation Campus, Oxfordshire OX11 0DE, U.K.

**Keywords:** ABC transport proteins, crystallography, molecular dynamics, thermodynamics

## Abstract

Under limiting sulfur availability, bacteria can assimilate sulfur from alkanesulfonates. Bacteria utilize ATP-binding cassette (ABC) transporters to internalise them for further processing to release sulfur. In gram-negative bacteria the TauABC and SsuABC ensure internalization, although, these two systems have common substrates, the former has been characterized as a taurine specific system. TauA and SsuA are substrate-binding proteins (SBPs) that bind and bring the alkanesulfonates to the ABC importer for transport. Here, we have determined the crystal structure of TauA and have characterized its thermodynamic binding parameters by isothermal titration calorimetry in complex with taurine and different alkanesulfonates. Our structures revealed that the coordination of the alkanesulfonates is conserved, with the exception of Asp205 that is absent from SsuA, but the thermodynamic parameters revealed a very high enthalpic penalty cost for binding of the other alkanesulfonates relative to taurine. Our molecular dynamic simulations indicated that the different levels of hydration of the binding site contributed to the selectivity for taurine over the other alkanesulfonates. Such selectivity mechanism is very likely to be employed by other SBPs of ABC transporters.

## Introduction

Bacteria are capable of thriving under both nutrient rich and limiting conditions. This extraordinary adaption is due to their ability of acquiring their essential nutrients via various mechanisms including outer membrane receptors and porins, as well as highly specific transporters in the inner membrane. In the absence of high levels of essential nutrients, both gram-positive and gram-negative bacteria utilize ATP-binding cassette (ABC) transporters (importers). ABC importers are energized by the binding and hydrolysis of ATP but they also require the presence of a substrate-binding protein (SBP) that usually have very high specificity and affinity for their substrates [1]. In gram-positive bacteria, the SBPs are usually tethered to the membrane whereas in gram-negative they are found in the periplasmic space [2]. Their role is usually to bind the scarcely available substrates and bring them to the transporter for internalization in the cytoplasm for either further processing or direct utilization.

An essential molecule for bacterial survival and growth is sulfur. Under nutrient rich conditions, bacteria assimilate sulfur either from cysteine or inorganic sulfate compounds [3]. Under sulfur starvation, bacteria rely on organic sulfur compounds such as sulfonates, sulfamates and sulfate esters as the sulfur source for growth. Bacteria have developed several systems to utilize alkanesulfonates for sulfur acquisition. In *Escherichia coli*, two operons that encode the organic sulfonate sulfur assimilation system, *tauABCD* and *ssuEADCB*, have been identified which are up-regulated only during sulfur starvation [3]. The *tauABCD* cluster is involved in sulfur assimilation from taurine (2-aminoethanesulfonic acid) where the *ssuEADCB* cluster encodes a sulfur utilization system that enables bacterial cells to assimilate sulfur from a broad range of alkanesulfonates except for taurine, such as 4-(2-hydroxyethyl)-1-piperazineethanesulfonic acid (HEPES), 3-(N-morpholino) propanesulfonic acid (MOPS) and piperazine-N, N-bis (2-ethanesulfonic acid) (PIPES). Taurine is one of the few naturally occurring alkanesulfonates [4] and forms the main component of bile. Taurine is widely distributed in animal tissues such as the large intestine, which constitutes the natural habitat of *E. coli* [3]. Animals are unable to metabolize taurine [4] and excess taurine is excreted and oxidized by bacteria to complete the natural redox cycle of sulfur. Disruption of the *tauABC* genes results in the loss of the ability to utilize taurine as a source of sulfur but does not affect the utilization of a range of other aliphatic sulfonates as sulfur sources [5].

In *E. coli*, the *ssuABC* and *tauABC* gene products encode for an ABC transporter for the uptake of alkanesulfonates and taurine, respectively; TauA is the SBP that recognizes and binds taurine, TauBC is the ABC transporter that mediates the transport of taurine in an ATP-dependent manner. The TauBC transporter belongs to the ABC importer family whereas TauC is the transmembrane domain (TMD) and TauB the nucleotide-binding protein (NBD) that binds and hydrolyses ATP for substrate import across the inner membrane. The alkanesulfonates that are imported by the SsuABC transporter are desulfonated by SsuD, a reduced flavin mononucleotide (FMN)-dependent monooxygenase [6]; the FMN is supplied by the NAD(P)H:FMN oxidoreductase SsuE, whereas release of sulfur from taurine is catalyzed by the α-ketoglutarate-dependent dioxygenase TauD [7].

The SsuA and TauA proteins belong to the class II SBPs that are characterized by two globular domains, linked by a flexible linker, that form a cleft where the substrate binds [2]. The crystal structures of SsuA from *E. coli* in its apo [8] and SsuA from *Xanthomonas axonopodis pv. citri* bound to alkanesulfonates [9] have been determined. In the apo state, the protein exists in an open conformation with both domains freely moving and rotating around the hinge. Upon ligand binding, the two domains pack tightly around the ligand and the SBP exhibits a closed state. Although, both the SsuABC and TauABC transporters share some substrates, they are also involved in the transport of distinct alkanesulfonates. It is unclear how substrate selectivity is achieved between the two systems.

In this study, we have determined the crystal structure of TauA from *E. coli* in complex with taurine. Site-directed mutagenesis indicates the importance of specific residues in the taurine binding pocket. Although, SBPs are usually described as highly specific for their substrate, we show that TauA is capable of binding different taurine analogues and alkanesulfonates, including phosphonate analogues, with different affinities. Comparison with the structure of SsuA revealed the molecular basis for substrate specificity in alkanesulfonate-binding proteins. In light of the binding data and the high-resolution crystal structures with different analogues, we propose that specificity and selectivity is due to desolvation of the binding site of TauA in addition to specific interactions with taurine. These observations are consistent with our molecular dynamics (MD) study of the hydration properties of the binding site of TauA bound to the different analogues. Analysis of other SBP high-resolution structures allows us to propose that the desolvation of SBPs is a rather universal mechanism for specificity and selectivity that is not unique to TauA.

## Materials and methods

### Protein expression and purification

The gene encoding for the mature TauA from *E. coli* (accession number: K12-Q47537) was cloned into the pEHisTEV vector. BL21 (DE3) cells transformed with pEHisTEVtauA plasmid were grown at 310 K. Isopropyl β-d-1-thiogalactopyranoside was added to a final concentration of 0.5 mM when the culture reached an OD_600_ of 0.6, and the growth continued at 298 K for 18 h. Cells were harvested by centrifugation (6000×***g***) and resuspended in PBS supplemented with 20 mM imidazole and 3 mM MgCl_2_. Cells were lysed by sonication (10 cycles; 10 s per cycle with 10 s interval on ice) followed by centrifugation at 30 000 g for 1 h. The supernatant was subjected to Ni-NTA affinity chromatography followed by overnight dialysis with TEV protease and size exclusion chromatography using a Superdex S-200 column equilibrated with 20 mM Tris (pH 7.5) and 150 mM NaCl. The size exclusion chromatography showed a monodisperse peak corresponding to TauA. SDS–PAGE indicated high purity.

### Site-directed mutagenesis

Alanine-substituted TauA mutants (Gln30Ala, Glu106Ala, Thr132Ala and Asp205Ala) were generated by PCR using the QuikChange Lightning Site-Directed Mutagenesis Kit (Agilent Technologies). Mutations were confirmed by DNA sequencing. Mutants were expressed and purified as for the wild-type TauA.

### Isothermal titration calorimetry

Binding of ligands to TauA and mutants was measured at 298 K using ITC (MicroCal iTC_200_ microcalorimeter, Malvern Instruments). The protein buffer was in 20 mM Tris (pH 7.5) and 150 mM NaCl (ITC buffer). Protein and ligands were diluted to desired concentrations using ITC buffer. The pH of protein and ligand was adjusted at 277 K. An amount of 200 µl of protein and 60 µl of ligand was added into the sample cell and injection syringe, respectively. Seventeen 2.4 µl injections were performed with a pre-injection of 0.5 µl, a 180 s interval and an injection speed of 0.5 µl/s. Control experiments were performed where ligand was injected into ITC buffer. The heat of interaction was obtained by subtracting control measurements (heat of dilution) from the experimental measurements. The baseline was generated and the concentration-normalized binding isotherm was integrated using the MicroCal Origin software (version 7). The first injection peak was deleted from the isotherm. The isotherm was fitted to a single-site binding model. Values of binding constant (*K*_a_, M^−1^), enthalpy change (Δ*H*, J/mol) and binding stoichiometry (n) were allowed to vary (unless mentioned specifically) during the fitting procedure. The dissociation constant (*K*_d_, M) and entropy change (Δ*S*, J/mol/K) were derived from *K*_a_, Δ*H* and n accordingly. The *K*_d_ value is derived from the absolute value of *K*_a_ (*K*_d_ = 1/K_a_).

### Crystallization of TauA in complex with ligands

Purified TauA at a concentration of 40 mg/ml was incubated with 1.25 mM taurine at 293 K for 1 h prior to crystallization. The taurine-bound TauA was crystallized in 0.2 M sodium iodide, 0.1 M Bis-Tris Propane (pH 8.5) and 20% (w/v) PEG 3350 by the sitting drop vapour diffusion method at 277 K. Upon optimization, the best crystals grew in 0.25 M sodium iodide, 0.1 M Bis-Tris propane (pH 8.5) and 24% (w/v) PEG 3350 at 293 K. The crystals appeared after 5 h and grew to maximum size in 24 h. To obtain the 2-Aminoethylphosphonic acid (2-AEP) and ACES complexes, TauA was (20 mg/ml) incubated with 10 mM 2-AEP, ACES or MES on ice for 6 h prior to crystallization. Crystals appeared in the same optimized conditions as the taurine complex after 2 days and grew to maximum size after a week.

### Data collection and structure determination

Crystals were cryoprotected by transferring the crystals in the crystallization buffer supplemented with 20% glycerol (v/v) and flash-cooled in liquid nitrogen for data collection. X-ray data were collected at beamlines I03, I23 and I24 at the Diamond Light Source. All crystals were indexed in space group *P*2_1_2_1_2_1_. Data collection statistics for taurine, 2-AEP, ACES and MES complexes are summarized in Supplementary Table S1. Since the crystals grew in high iodine salt conditions, initial phases were calculated from a crystal collected at the long wavelength beamline I23 [10] close to the iodine L-III edge, 2.37 Å, at 1.77 Å resolution. In total 360° of data were collected using the inverse-beam method (10° wedges) (Supplementary Table S1). Three datasets were collected from one crystal. Data were merged and scaled using XSCALE-XDS [11]. Data were processed with the program suite SHELEXC/D/E [12], assuming a solvent content of 45% and eight iodine sites in the asymmetric unit. The initial phases were subjected to Buccaneer [13] and 83.4% of the initial model was built. The current model was used as a search model for molecular replacement of the TauA-taurine structure in Phaser [14]. Iterative cycles of model building and refinement were completed using Phenix [15] and COOT [16]. Peaks of anomalous maps (contoured at 8 σ) suggested the presence of a sulfur atom from taurine at the binding pocket and iodine atoms at the surface of TauA protein, respectively. Water molecules were automatically added by ARP/wARP [17]. The final TauA-taurine structure was refined to 1.30 Å with an R-factor of 14.5% and R-free of 18.3% (Supplementary Table S1). Structures of TauA-2-AEP (1.62 Å), TauA-ACES (1.5 Å) and TauA-MES (1.55 Å) were solved by molecular replacement using the refined TauA-taurine structure as a search model. Refinement statistics for all complexes are summarized in Supplementary Table S1.

The structure factors and co-ordinates have been deposited to the Protein Data Bank with accession numbers: 6STL (TauA-taurine), 6SSY (TauA-2-AEP), 6ST0 (TauA-ACES), 6ST1 (TauA-MES).

### Molecular dynamics simulations

MD simulations were performed on ligand-free and on the various substrate-bound forms of TauA analyzed in this work to provide information on the hydration properties of the protein [18]. For the ligand-free form, we used the TauA-taurine crystal structure depleted of the ligand. This model is representative of the bound conformation of the protein in the absence of ligands. While this is not fully representative of the apo form of TauA, whose structure is not available, it is optimal to compare the role of waters with the various ligand-bounds states, which are all in the same closed conformation of the protein.

MD simulations were set as previously described [19] by using the GROMACS [20] package by using the all-atom AMBER99sb [21] force field in combination with the TIP4P-ew explicit water model [22]. The latter was specifically optimized for MD simulations performed with the use of Ewald summation methods, and showed extremely good agreement with experimental data over a range of temperatures. To avoid any bias on the hydration status of the protein derived from the MD analyses, crystallographic water molecules were removed from the starting models. The simulations were carried out in the NPT ensemble with periodic boundary conditions at a constant temperature of 300 K and the constant pressure of 1 atm. A rectangular box was used to accommodate the protein/ligand, water molecules, and ions. Number of atoms/waters and box sizes are reported in Table 3.

## Results

### Structure of TauA in complex with taurine

**Figure 1.**
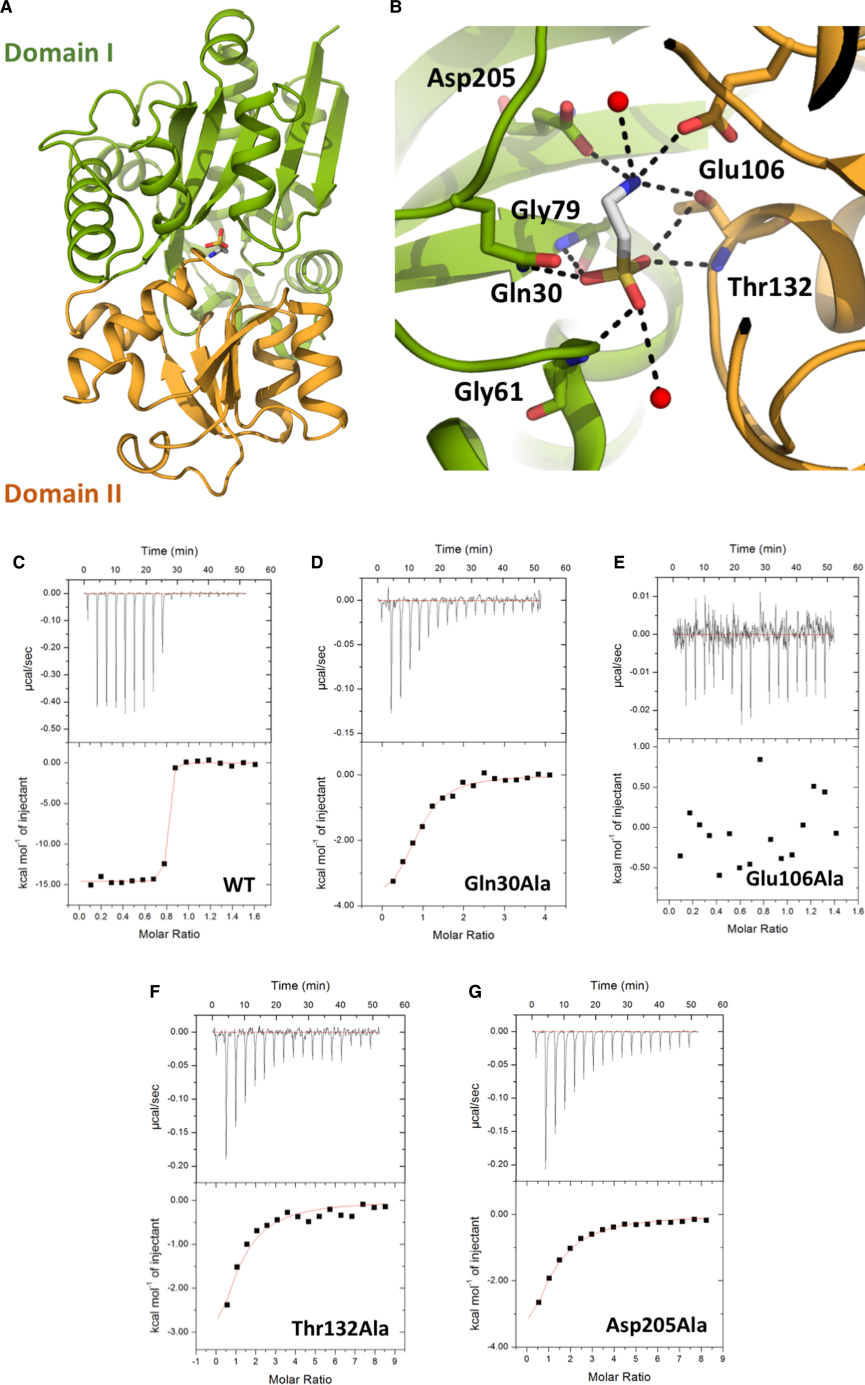
TauA crystal structure in complex with taurine and substrate binding site characterization. (**A**) Cartoon representation of TauA in complex with taurine. Taurine is shown in sticks; carbon atoms are shown in gray, oxygen in red, nitrogen in blue and sulfur in yellow. TauA displays a characteristic class II SBP structure with two globular domains, I and II, linked by a flexible linker; domain I is coloured green and domain II orange. Taurine binds in the cleft formed by the two domains. (**B**) Close-up view of the binding site. Taurine is bound by residues from both domains. Coordinating oxygens are shown as red spheres and hydrogen-bonds as dashed lines. Van der Waals interacting side chains have been omitted for clarity. (**C**–**G**) Calorimetric titration taurine binding to TauA and mutants (see also Table 1). Each peak (top panel) represents an injection of 2.4 µl of taurine into 200 µl of wild-type TauA or TauA mutant. The bottom panel shows the integrated heat obtained from the raw data, after subtracting the heat of dilution. Binding of taurine to TauA is exothermic with a binding affinity of 1.6 nM. All the TauA mutants show a decrease in taurine-binding affinity whereas the Glu106Ala mutation is detrimental to taurine binding.

The crystal structure of TauA from *E. coli* in complex with taurine was determined by iodide single-wavelength anomalous diffraction (SAD) at 2.37 Å and refined to a resolution of 1.3 Å (Figure 1 and Supplementary Table S1). The space group was determined to be *P*2_1_2_1_2_1_ with two copies of TauA in the asymmetric unit. The overall structure of TauA resembles other SBPs, including SsuA from *E. coli* and *X. citri* [9]. The *E. coli* TauA shares 23% sequence identity (41% similarity) with the *E. coli* SsuA and 22% identity (34% similarity) with the *X. citri* SsuA. Despite their limited sequence identity, their overall structure is very similar (Supplementary Figure S1). TauA is composed of two α/β-domains (domain I (23–104 and 202–319) and domain II (105–201)) connected by two-loop hinge regions (103–106 and 200–202) with the substrate-binding site located at the interface of the two domains (Figure 1); both domains are composed of five β-strands flanked by 11 and 4 α-helices, respectively.

**Table 1:** Thermodynamic parameters for taurine binding to TauA and mutants

	*K*_d_ (nM)	Stoichiometry	Δ*H* (kJ/mol)	Δ*S* (J/mol/K)
WT	1.6	0.768(±0.38%)	−61.04 (±0.08%)	−3.05
Gln30Ala	3480	0.83(±6%)	−17.87 (±8%)	3.72
Asp106Ala	NB	NB	NB	NB
Thr132Ala	20 620	0.8*	−28.76 (±8%)	−0.54
Asp205Ala	22 880	0.8*	−34.83 (±2%)	−2.34

NB, no binding.

* Stoichiometry fixed to 0.8 during data fitting.

Inspection of the electron density maps identified strong electron density for taurine (Supplementary Figure S2). An anomalous difference map was also calculated at 2.37 Å and further verified the presence of the taurine sulfur atom (Supplementary Figure S2). The taurine binding site is composed of residues from both domains (Figure 1). The sulfonate group is co-ordinated by hydrogen bonds with the side chain of Gln30 and the protein main chain amine groups of Gly61, Gly79 and Thr132. The taurine ethylamine group is hydrogen bonded by the side chains of Glu106, Thr132 and Asp205. Taurine is further stabilized in the binding site of TauA by van der Waals interactions. We could not obtain any crystals for the apo TauA suggesting flexibility between the two domains; the *E. coli* SsuA is a closer sequence homologue to the *E. coli* TauA (sequence identity of 23% and similarity of 41%) and the superimposition of the taurine-bound TauA with apo SsuA from *E. coli* [8] resulted in an rmsd of 3.15 Å over 176 Cα atoms; the apo SsuA exists in an open conformation where the two domains are separated from each other and the substrate-binding pocket is accessible for ligand binding. The taurine-bound TauA from *E. coli* can be superimposed with the MES-bound SsuA from *X. citri* [9] with an rmsd of 1.60 Å over 162 Cα atoms (Supplementary Figure S1).

### Characterization of the TauA binding site

Based on the interactions from the crystal structure of TauA with taurine, mutants were designed in order to further characterize the role of specific amino acids on their role in taurine binding. The binding affinity of wild-type TauA and mutants, Gln30Ala, Glu106Ala, Thr132Ala and Asp205Ala, for taurine were determined by isothermal titration calorimetry (ITC) (Figure 1 and Table 1). TauA binds to taurine with an apparent affinity of 1.6 nM. Loss of the hydrogen bond between Gln30Ala and the sulfonate group of taurine reduced its affinity to 3.5 µM, whereas loss of the hydrogen bond between the ethylamine group of taurine and Thr132Ala and Asp205Ala resulted in reduction in the affinity to 20.6 µM and 22.9 µM, respectively. No binding affinity could be measured for the Glu106Ala mutant. The binding data suggest that the interaction of the sulfonate group with TauA partly mediates its recognition but the key determinant for binding appears to be the interaction between Glu106Ala and ethylamine. Indeed, Glu106 is found in one of the hinges, and this mutation is detrimental in providing a stable closed TauA conformation.

### TauA substrate specificity

**Figure 2.**
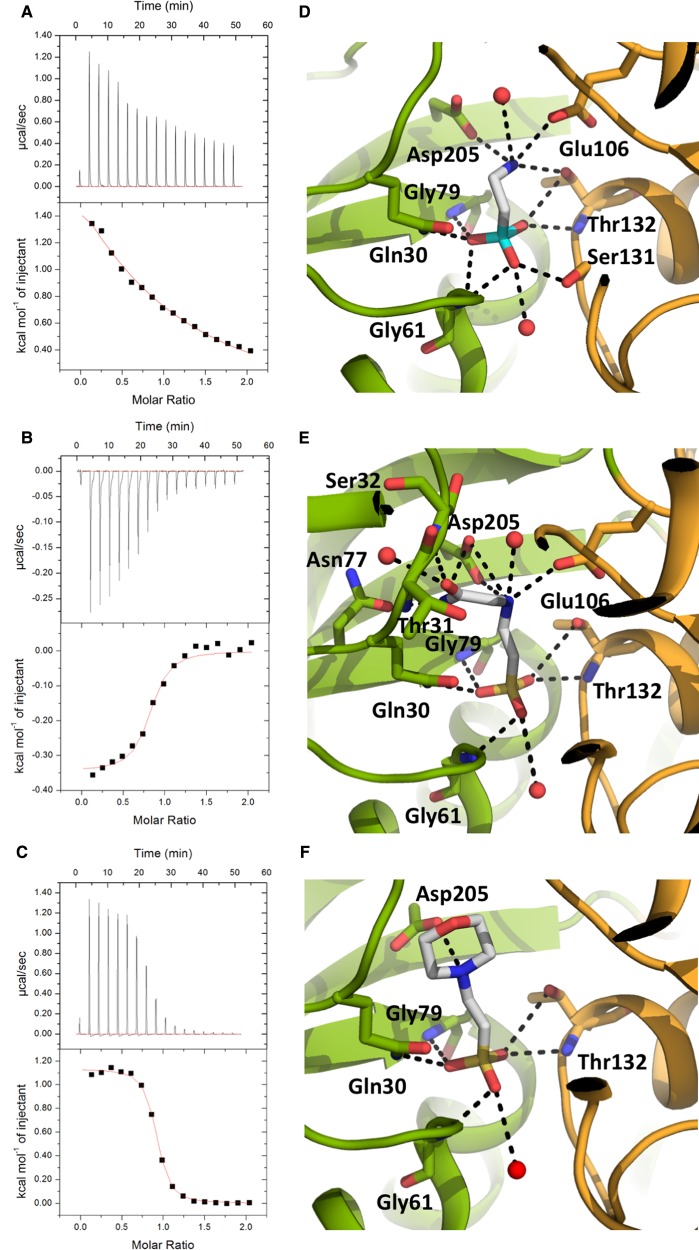
TauA in complex with different alkanesulfonate analogues. (**A**–**C**) Calorimetric titration taurine binding to TauA and alkanesulfonate derivatives (see also Table 2). Each peak (top panel) represents an injection of 2.4 µl of taurine into 200 µl of TauA. The bottom panel shows the integrated heat obtained from the raw data, after subtracting the heat of dilution. Binding of 2-AEP (**A**) and MES (**B**) to TauA is endothermic whereas binding of ACES (**C**) is exothermic. (**D**–**F**) Close up views of TauA in complex with the alkanesulfonate analogues 2-AEP (**D**), ACES (**E**) and MES (**F**). All compounds show similar interactions to taurine with the exception of 2-AEP that requires Ser131 to adopt a different rotamer to co-ordinate the smaller phosphonate group. The colour scheme is the same as in Figure 1; the phosphate group of 2-AEP is shown in cyan.

Unlike SsuA that can transport various alkanesulfonates, TauA is usually referred to as a taurine-specific SBP, although ligand promiscuity has been reported [5], and we sought to investigate its specificity for other taurine structural analogues (alkanesulfonates) by ITC in order to investigate its specificity and also identify which functional groups of the taurine molecule are important for selectivity. 2-Aminoethylphosphonic acid (2-AEP) was selected as a structural analogue since it contains a phosphonate group instead of sulfonate. Although, 2-AEP has the same structural configuration as taurine, very weak binding could be measured, *K*_d_ of 649 µM, between TauA and 2-AEP (Figure 2 and Table 2). We also investigated if larger compounds such as the N-(2-acetamido)-2-aminoethanesulfonic acid (ACES) analogue, that has an acetamido group after the ethylamine group of taurine, and 2-(N-morpholino)ethanesulfonate (MES), that contains a morpholino group, could bind to TauA; we measured an apparent affinity of 7.4 µM and 2.8 µM, respectively (Figure 2 and Table 2). The binding data clearly indicate that the sulfonate group is important for recognition but larger compounds could be accommodated within the binding site, but with lower affinity.

**Table 2:** Thermodynamic parameters for taurine and alkanesulfonates binding to TauA

	*K*_d_ (nM)	Stoichiometry	Δ*H* (kJ/mol)	Δ*S* (J/mol/K)
taurine	1.6	0.768(±0.38%)	−61.04 (±0.08%)	−3.05
2-AEP	649 351	0.8*	0.016 (±0.03%)	2.33
ACES	7463	0.79(±2.4%)	−1.44 (±3.3%)	1.87
MES	2849	0.868(±0.5%)	4.74 (±0.8%)	2.45

* Stoichiometry fixed to 0.8 during data fitting.

In light of the binding data, we co-crystallized TauA with 2-AEP, ACES and MES and collected X-ray data at 1.62 Å, 1.5 Å and 1.55 Å resolution, respectively (Supplementary Table S1). Inspection of electron density maps revealed electron density for 2-AEP, ACES and MES (Figure 2 and Supplementary Figure S2). Despite the very low binding affinity between TauA and 2-AEP by ITC, a complex could be formed under crystallisation conditions. The 2-AEP is co-ordinated in a similar manner as taurine with an additional hydrogen bond between the phosphonate group and the side chain of Ser109. This hydrogen bond is absent from the taurine complex and it is a result of the different rotamer that Ser109 adopts in the presence of 2-AEP in order to co-ordinate the smaller phosphonate anion. The ACES with its longer acetamido group displays similar interactions as the taurine complex but its amine group is making additional hydrogen bonds to Asp84, whereas Asp84 in the taurine complex is bound to a water molecule, and its acetamido group is stabilized by hydrogen bonds between the side chain of Asn55 and the main chain amine of Ser10. In the taurine complex, the site occupied by the acetamido group is hydrated. In the MES complex, the morpholino group is stabilized by van der Waals interactions with Glu106 and Trp176 and a hydrogen bond from Asp205. Although, there is a discrepancy between the binding data and the crystals structures for both 2-AEP and ACES, it is apparent that TauA can discriminate between the different taurine analogues. SBPs have to undergo large conformational changes between the apo and substrate-bound states driven by ligand binding and in the case of 2-AEP, it is likely that 2-AEP binding that is also coupled to local conformational changes at the binding site, Ser109, is unfavourable, thus allowing the TauA to distinguish over smaller ligands. We also propose that desolvation of the binding site, loss of water molecules, contributes to the lower affinity for ACES and MES over taurine, although they display more hydrogen bonds than taurine.

### Molecular dynamics simulations

To further investigate the discrepancy in affinity of the TauA over a wide range of structurally similar compounds, we performed MD simulations of the protein in its unbound or bound states. MD of 100 ns were performed for each system by using the amber99SB force field in association with the TIP4P-ew water model (see Materials and Methods), a combination that has shown to be able to reproduce accurately the structure, dynamics and thermodynamics of water molecules at the surface of proteins [23,24] as well as to sample the protein and peptide conformations, as shown by cross-validation with NMR scalar couplings and relaxation [25]. The simulations were based on the experimental structures determined in this study, which were solvated by explicit waters into rectangular boxes (See Table 3 for statistics). Simulations run at 300 K and 1 atm showed significant stability of the protein structures, with steady RMSD values remaining within 1.1 Å in all the constructs. Similarly, other parameters such as gyration radius and secondary structure elements were retained along the trajectories by reproducing those of the starting crystallographic models.

**Figure 3.**
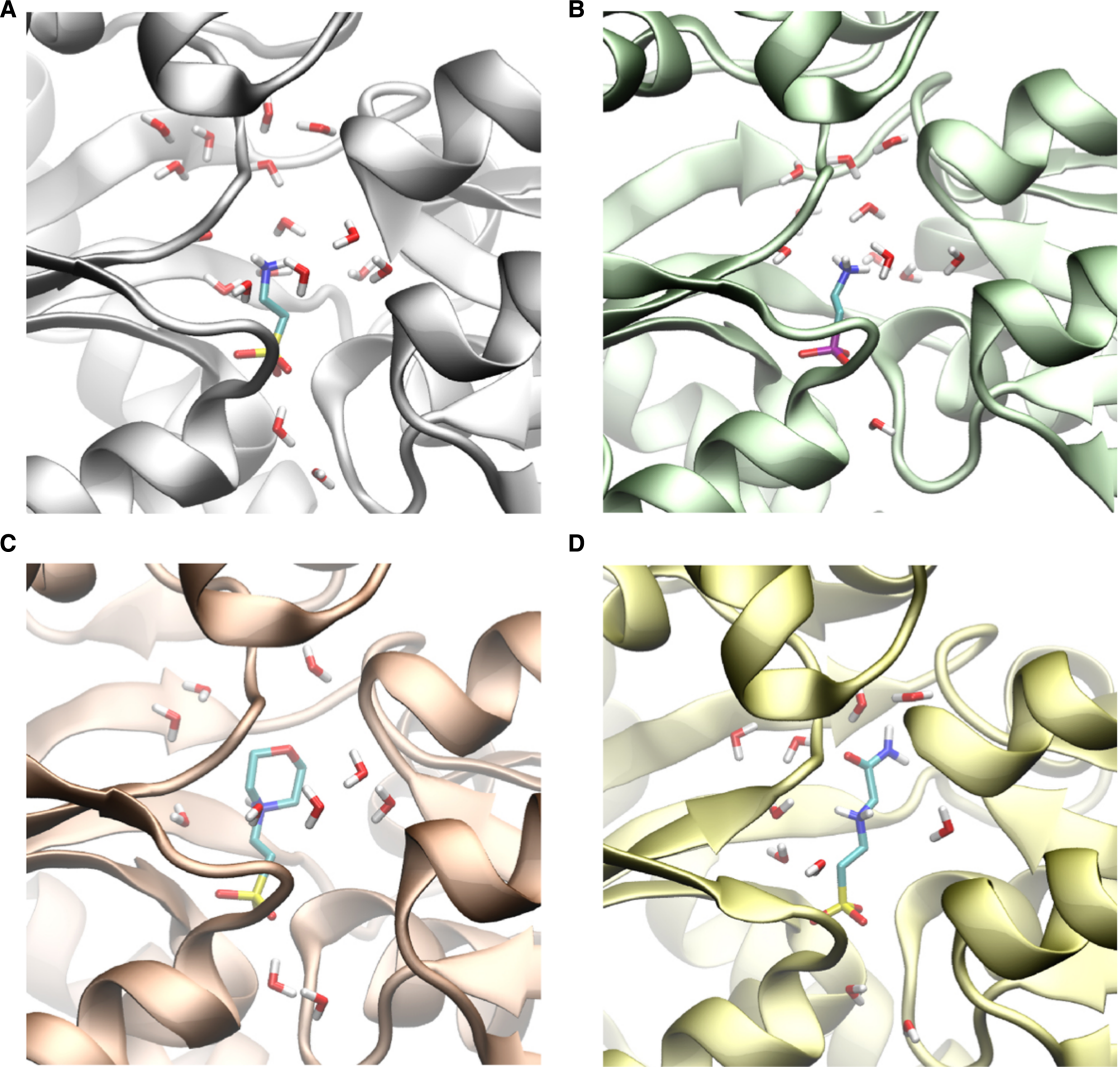
Hydration properties of TauA in complex with different alkanesulfonate analogues as analysed by MD simulations. (**A**–**D**) Snapshots of the simulations where selected to represent conformations having a number of waters in the cavity corresponding to the median of the distribution in the sampling. TauA in complex with taurine (**A**), 2-AEP (**B**), MES (**C**), ACE (**D**). The ligands are shown as sticks and colour scheme is the same as in Figures 1 and 2 with the exception of carbon that is shown in cyan.

In addition to all the complexes resolved, we also simulated the protein in its bound conformation but in the absence of ligands; while this conformation is not representative of the apo form of TauA, it is a convenient control simulation to study the hydration properties of the protein in the absence of ligands. In particular, we analyzed the MD sampling to characterize the stabilizing role of waters bound to the binding pocket of TauA in the binding of the structurally similar compounds. The results indicate the presence of a large number of water molecules in the binding cavity, in both bound and unbound states (Table 3). In particular, in the conformation of the bound state, the binding pocket is able to accommodate on average 20.2 water molecules (Table 3). In the presence of the ligands, however, not all these water molecules are expelled from the binding cavity, with some complexes retaining a large number of waters (Figure 3). Of these, the binding with taurine retains the largest number of waters (17.5 in average), whereas MES, ACE and AEP retain 10.2, 10.8 and 10.5 waters, respectively (Figure 3). Thus, the hydration properties of the cavity appear to vary across the different bound states, with the taurine complex showing distinctive hydration properties with respect to the others. The analysis of the potential energy indicates that bound waters in the TauA-taurine complex are the most stable in our simulations (−94.4 kJ/mol), and overcome the potential energy of bulk waters (−92.2 kJ/mol). Conversely, waters in the binding cavity of TauA bound to MES, ACE and AEP are destabilized with respect to the potential energy terms (−88.2, −86.1, −87.7 kJ/mol, respectively).

**Table 3:** Statistics of the MD simulations

System	Average number of waters	Average potential energy per water (kJ/mol)	Overall difference with bulk (kJ/mol)
No ligands	20.2	−93.4	0
Taurine	17.5	−94.4	+234.7
MES	10.2	−88.2	+987.0
ACES	10.8	−86.1	+956.8
2-AEP	10.5	−87.7	+965.83
Bulk Water		−92.2	
System	Total number of waters	Total number of atoms	Initial box volume nm^3^
Apo protein	11 058	48 783	381.08
Taurine	11 846	51 943	417.73
MES	10 824	48 379	377.69
ACES	10 879	48 153	378.79
2-AEP	11 065	48 827	376.11

Water properties in the binding site (top) and general parameters of the simulations (bottom).

Taken together these data suggest that the overall potential energy of bound waters in the TauA-taurine complex is the lowest of all the bound states analyzed here, with an overall difference being in the order of ∼700 kJ/mol. Of course, this contribution represents one energetic term and is likely partially counterbalanced by the entropic term of restricting the degrees of freedom of waters bound in the case of TauA-taurine binding. Nevertheless, we note the striking energy difference in the potential energy of bound waters in TauA-taurine with comparison to the other compounds having significantly lower affinity for TauA.

## Discussion

Although bacteria have two independent ABC-transporter-dependent sulfur assimilation pathways, TauABC and SsuABC, to import sulfur containing compounds, alkanesulfonates, inside their cells, there is some substrate overlap but also differences [5]. The alkanesulfonate taurine can only be transported by the TauABC and not the SsuABC transporter, suggesting differences in the SBP and ABC transporter. In contrast, SsuA is more promiscuous to its substrate recognition [5]. The structure of TauA revealed that its binding site is aligned by charged amino acids whereas the SsuA is more hydrophobic [9] and it is also aligned by several Gly and Ala residues, Gly186, Gly187, Ala13 and Ala42 (the equivalent residues in TauA are Leu204, Asp205, Ser32 and Ser60), that would allow it to bind a wider range of structurally different alkanesulfonates as a result of a bigger binding site with less steric clashes (Supplementary Figure S1). Asp205 appears to provide further restriction of the TauA binding site. Therefore, the variation of the binding pocket volumes establishes the structural basis of substrate specificity between TauA and SsuA.

Comparison of the sequence and crystal structures of TauA and SsuA reveal some conserved structural elements in ligand recognition and binding (Supplementary Figure S1). In both structures, the sulfonate group of taurine and alkanesulfonates contacts the protein via a cluster of conserved residues including glutamine, glycine, serine, and threonine(TauA)/serine(SsuA) (Supplementary Figure S1). Selectivity for taurine by the TauA is due to the side chain of Asp205 that co-ordinates binding of the ethylamine group (Figure 1) that is absent from SsuA, the equivalent residue is Gly187 (Supplementary Figure S1). Although, mutagenesis of Asp205Ala did not abolish binding, it reduced the affinity of TauA for taurine to 22.8 µM from 1.6 nM suggesting a key role in coordinating taurine binding (Table 1). Selectivity will be further conferred by the TMD domain of their respective ABC transporters, TauBC ans SsuBC.

TauABC has been shown to transport several alkanesulfonates with preference to taurine [5]. We determined the binding constants and structures of TauA in complex with several taurine analogues in order to deduce the molecular mechanism of substrate promiscuity as well as preference for taurine. Interestingly, our ITC data revealed that TauA can bind taurine with the highest affinity, 1.6 nM, relative to the alkanesulfonates ACES, 7.4 µM, and MES, 2.8 µM. The sulfonate group is not a strict determinant for selectivity as it could bind a phosphonate analogue 2-AEP but with much weaker affinity, 0.64 mM. The crystal structure of TauA with 2-AEP suggests that although the phosphonate group has the same tetrahedral coordination as sulfonate, it cannot be effectively co-ordinated in the binding site and that the ethylamine group alone is not sufficient to co-ordinate tight binding of 2-AEP to taurine. The internuclear distance for the sulfate and phosphate anions has been determined to be 0.38 nm for S-O and 0.36 nm for P-O [26] suggesting that the smaller 2-AEP phosphonate ionic radius results in weaker interactions with the binding site of TauA. The crystal structures of TauA with 2-AEP and ACES appear to be in ‘disagreement’ with the ITC data since the structures show that these molecules have formed more hydrogen bonds relative to taurine, they display much weaker affinities. The thermodynamic parameters determined from ITC (Table 2) suggest that the enthalpy Δ*H* is significantly altered from the binding of the different analogues whereas the entropy Δ*S* is slightly destabilized. Comparing the ΔΔ*S* between the taurine and the different analogues, it becomes apparent that all compounds are marginally destabilized to a similar extend upon binding with an average ΔΔ*S* of 5 J/mol/K. In contrast, the contribution of the enthalpy to the binding is significantly altered (Table 2) with the ΔΔ*H* difference between taurine and the other alkanesulfonates being over 60 kJ/mol. Although, the Δ*H* parameter can provide some explanation on the selectivity, it is important to note that SBPs have to undergo significant conformational changes between their apo and substrate-bound state, therefore significantly contributing to the thermodynamic parameters; in complex with taurine and 2-AEP, the interactions, including water molecules, are nearly identical with the exception of Ser131 that has to flip to bind the phosphonate group. This conformational change can also attribute to the positive Δ*H* for 2-AEP. The higher enthalpic penalty can be attributed to both the desolvation of the binding cavity and the intermolecular interface upon closure of the domains I and II when a substrate binds.

Our high-resolution structures have allowed us to identify conserved and non-conserved water molecules within the binding cavity (Supplementary Figure S3) and investigate their contribution to ligand selectivity by MD. Our structures show that upon ligand binding some waters have to be displaced to accommodate the larger alkanesulfonates such as ACES and MES. From this comparison, it is evident, that desolvation of the binding site will contribute towards the higher enthalpy penalty as measured by ITC. Analysis of the thermodynamic parameters from ITC can only provide us with global protein behaviour. Although Δ*H* is becoming more positive, that could be attributed to desolvation of the TauA upon ligand binding, ITC cannot provide accurate details on desolvation of the binding site alone since it measures the contribution of water exclusion upon domain I and II closure in the presence of ligands. In order to gain a better understanding on the contribution of desolvation of the binding cavity and its contribution to ligand selectivity, we performed MD simulations. The simulations were in a very close agreement with the ITC data that desolvation has a very high penalty score. Binding of the taurine analogues resulted in the displacement of around 10 water molecules compared with three water molecules in the presence of taurine, which is reflective by the large difference in total potential energy. Despite the high structural similarity between taurine and 2-AEP, the later also displaces 10 water molecules. The main difference in these two bound states is associated with the mobility of 2-AEP in the cavity compared with that of taurine. Taurine is indeed strongly anchored to the protein via its sulfonate group whereas the anchoring of 2-AEP via the phosphonate group is loose, leading to some sweeping effects by the amine region of 2-AEP that disrupts the water network in the cavity and reduces the overall hydration in this complex.

Desolvation has previously been reported as a determinant of ligand affinity in proteins and it has been suggested that in SBPs it may also contribute to substrate binding. From the structures and ITC data of the oligopeptide binding protein OppA from *Lactococcus lactis* [27], it has been proposed that enthalpy and entropy are determinants of different oligopeptide binding as a function of desolvation. The relative changes of ΔΔ*H* of OppA for the different peptides are in the range of 1–10 kJ/mol with displacement of one or three water molecules, for bulkier side chains, upon binding of the different peptides [27]. This is in contrast with TauA with ΔΔ*H* differences in the range of 60 kJ/mol and displacement of at least 10 water molecules for all alkanesulfonates from the MD simulations suggesting a more intricate mechanism of selectivity. A recent study of the sialic acid SBP SiaP from *Haemophilus influenzae* showed that mutations in the binding site can alter the affinity of the substrate from 30 nM to 42 µM, with a ΔΔ*H* of 50 kJ/mol, due to the disruption of the water network coordination [28]; this value is very similar to the observed ΔΔ*H* changes in TauA suggesting that desolvation as a function of water network disruption is a key determinant is ligand selectivity. In light of our data, we propose that the SBPs for sulfur assimilation can distinguish between the different analogues as a function of binding site desolvation but the high affinity of taurine also requires a single specific amino acid, Asp205, that is not conserved between the two different subclasses. Although, this observation has not previously been reported for SBPs, it is likely to be applicable to other systems.

## Abbreviations

2-AEP: 2-Aminoethylphosphonic acid
ABC: ATP-binding cassette
ACES: N-(2-acetamido)-2-aminoethanesulfonic acid
FMN: flavin mononucleotide
HEPES: 4-(2-hydroxyethyl)-1-piperazineethanesulfonic acid
ITC: isothermal titration calorimetry
MD: molecular dynamics
MES: 2-(N-morpholino)ethanesulfonate
MOPS: 3-(N-morpholino) propanesulfonic acid
NBD: nucleotide-binding protein
SAD: single-wavelength anomalous diffraction
SBP: substrate-binding protein
TMD: transmembrane domain
