# Supplementary material for: Desolvation of the substrate-binding protein TauA dictates ligand specificity for the alkanesulfonate ABC importer TauABC
Source: Biochem J. 2019 Dec 10;476(23):3649–60. doi: 10.1042/BCJ20190779 (PMC6906117; doi:10.1042/BCJ20190779)
Supplement: Supplementary Table S1 and Figures S1-S3 [file BCJ-476-3649-s1.pdf]

Table S1. Data collection and refinement statistics. Values in parentheses are for the highest-resolution shell.

	**TauA-taurine (phasing)**	**TauA-taurine (refinement)**	**TauA-2-AEP**	**TauA-ACES**	**TauA-MES**
**Data collection**					
Beamline	DLS-I23	DLS-I03	DLS-I24	DLS-I24	DLS-I24
Wavelength (Å)	2.3751	0.9763	0.9686	0.9686	0.9686
Resolution (Å)	65.47-1.77 (1.81-1.77)	67.53-1.30 (1.33-1.30)	65.68-1.62 (1.65-1.62)	35.9-1.50 (1.53-1.50)	40.97-1.55 (1.59 – 1.55)
Space group	$P2_12_12_1$	$P2_12_12_1$	$P2_12_12_1$	$P2_12_12_1$	$P2_12_12_1$
Cell dimensions					
a,b,c (Å)	45.01, 71.32, 165.03	44.96, 74.09, 164.11	45.1, 71.64, 164.55	45.16, 71.8, 164.78	45.22, 73.0, 163.90
α, β, γ (°)	90, 90, 90	90, 90, 90	90, 90, 90	90, 90, 90	90, 90, 90
Total reflections	873204 (39274)	417657 (20677)	433767 (19645)	330516 (14581)	362377 (24578)
Unique reflections	45258 (2202)	130542 (6414)	68655 (3375)	85811 (4180)	77044 (5553)
Completeness (%)	86.0 (74.0)	97.5 (96.9)	99.8 (99.9)	98.9 (97.8)	97.0 (95.3)
Anomalous Completeness (%)	85.2 (72.8)	-	-	-	-
Redundancy	19.3 (17.8)	3.2 (3.2)	6.3 (5.8)	3.9 (3.5)	4.7 (4.4)
Anomalous Redundancy	9.9 (9.1)	-	-	-	-
Rmerge	0.12 (0.83)	0.056 (0.43)	0.1 (0.86)	0.072 (0.8)	0.09 (1.22)
I/sig(I)	15.9 (3.1)	11 (2.5)	10.9 (1.9)	11.9 (1.7)	8 (1)
CC1/2	1 (0.8)	1 (0.8)	1 (0.6)	1 (0.5)	1 (0.5)
**Refinement**					
R_{work}/R_{free} (%)	-	14.5/18.3	16.8/20.3	15.6/22.2	19.3/23.1
Average B-factors (Å^2)	-	16.6	20.8	20.9	24.9
protein	-	15.1	20.1	19.8	24.1
taurine	-	18.3	26.7	22.7	31.3
Ramachandran (%)					
Favoured	-	98.48	98.48	98.65	97.8
Allowed	-	1.18	1.18	1.02	1.86
Outliers	-	0.34	0.34	0.00	0.34

R-free was calculated using 5% of data excluded from refinement.

(A)

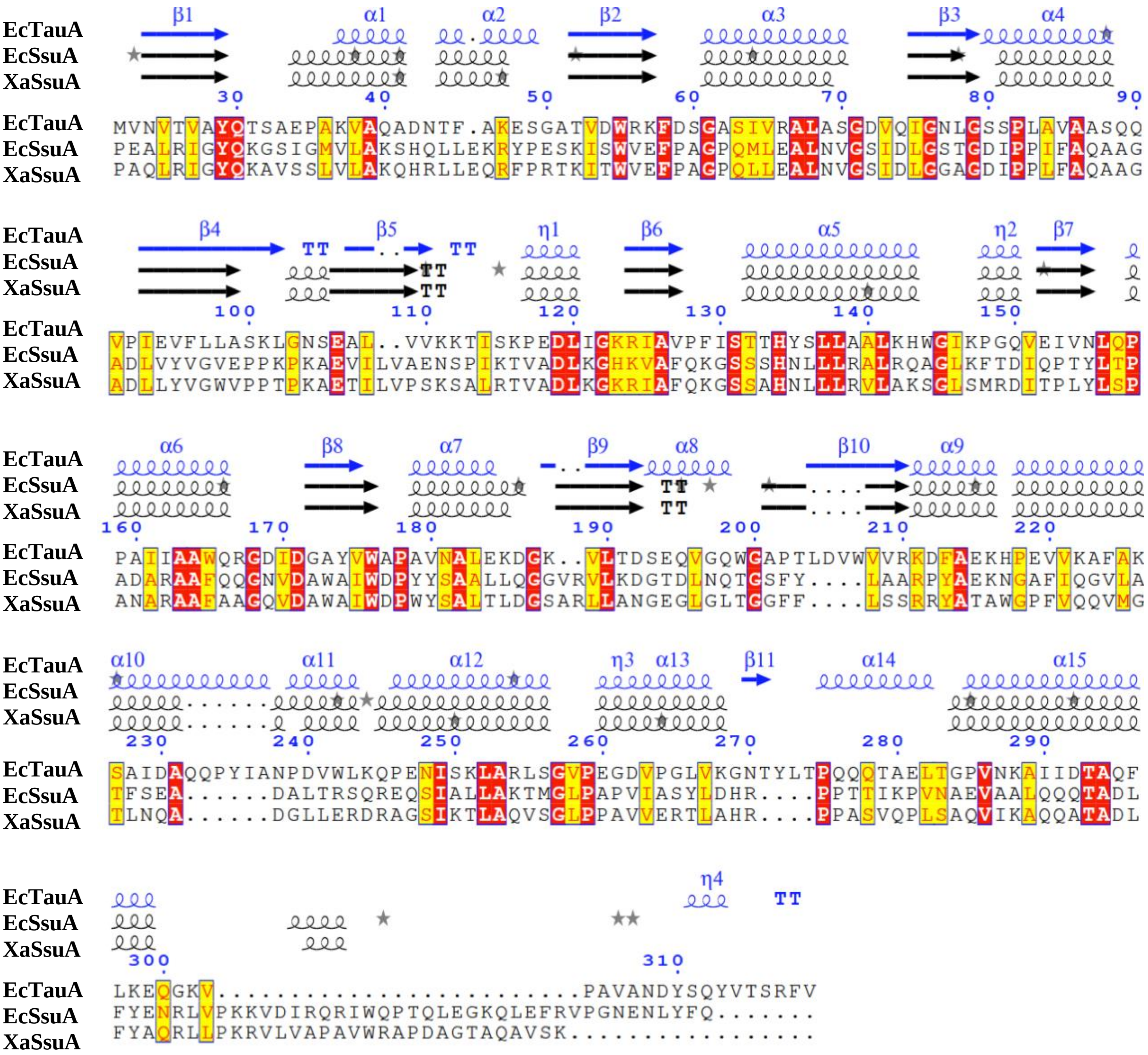
EcTauA
EcSsuA
XaSsuA
β1 α1 α2 β2 α3 β3 α4
30 40 50 60 70 80 90
EcTauA MVNVTVAYQTSAEPAKVAQADNTF.AKESGATVDWRKFDSGASIVRALASGDVQIGNLGSSPLAVAASQQ
EcSsuA PEALRIGYQKGSIGMVLAKSHQLLEKRYPESKISWVEFPAGPQMLEALNVGSIDLGSTGDIPPIFAQAAG
XaSsuA PAQLRIGYQKAVSSLVLAKQHRLLEQRFPRTKITWVEFPAGPQLLEALNVGSIDLGGAGDIPPLFAQAAG
β4 TT β5 TT η1 β6 α5 η2 β7
100 110 120 130 140 150
EcTauA VPIEVFLLASKLGNSEAL..VVKKTISKPEDLIGKRIAVPFISTTHYSLLAALKHWGIKPGQVEIVNLQP
EcSsuA ADLVYVGVEPPKPKAEVILVAENSPIKTVADLKGHKVAFQKGSSSHNLLLRALRQAGLKFTDIQPTYLTP
XaSsuA ADLLYVGWVPPTPKAETILVPSKSALRTVADLKGKRIAFQKGSSAHNLLLRVLAKSGLSMRDITPLYLSP
α6 β8 α7 β9 α8 β10 α9
160 170 180 190 200 210 220
EcTauA PAIIAAWQRGDIDGAYVWAPAVNALEKDGK..VLTDSEQVGQWGAPTLDVWVVRKDFAEKHPEVVKAFAK
EcSsuA ADARAAFQQGNVDAWAIWDPYYSAALLQGGVRVLKDGTDLNQTGSFY....LAARPYAEKNGAFIQGVLA
XaSsuA ANARAAFAAGQVDAWAIWDPWYSALTLDGSARLLANGEGLGLTGGFF....LSSRRYATAWGPFVQQVMG
α10 α11 α12 η3 α13 β11 α14 α15
230 240 250 260 270 280 290
EcTauA SAIDAQQPYIANPDVWLKQPENISKLARLSGVPEGDVPGLVKGNTYLTPQQQTAELTGPVNKAIIDTAQF
EcSsuA TFSEA......DALTRSQREQSIALLAKTMGLPAPVIASYLDHR....PPTTIKPVNAEVAALQQQTADL
XaSsuA TLNQA......DGLLERDRAGSIKTLAQVSGLPPAVVERTLAHR....PPASVQPLSAQVIKAQQATADL
η4 TT
300 310
EcTauA LKEQGKV.......................PAVANDYSQYVTSRFV
EcSsuA FYENRLVPKKVDIRQRIWQPTQLEGKQLEFRVPGNENLYFQ.......
XaSsuA FYAQRLIPKRVLVAPAVWRAPDAGTAQAVSK.................

(B)

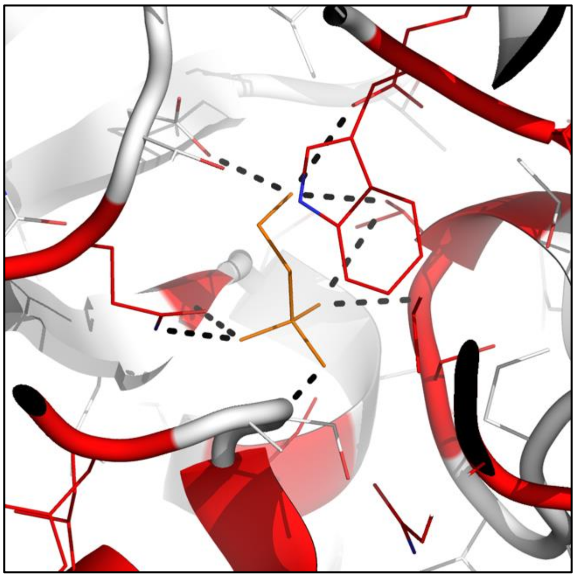

(C)

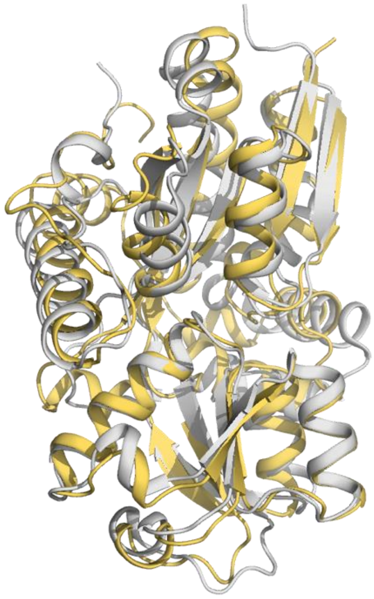

(D)

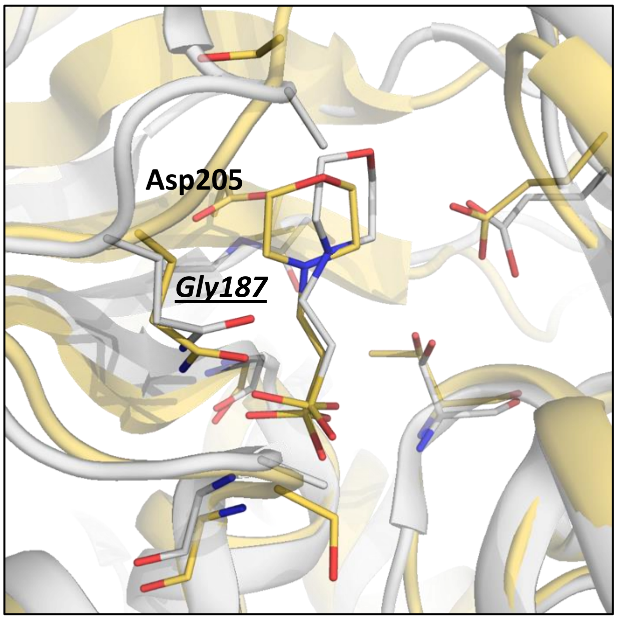

Figure S1. Sequence and structural comparison of TauA and SsuA. (A) Sequence alignment of *E. coli* TauA with *E. coli* and *X. axonopodis* SsuA, respectively. Conserved amino acids are shown in red boxes. Sequence alignment was performed with Clustal Omega. (B) Close-up of the *E. coli* TauA bidning site with the conserved residues from the sequence alignment mapped onto the TauA-taurine structure. Conserved residues are shown in red and non-conserved in grey. The taurine is coordinated by conserved and non-conserved residues. (C) Superimposition of *E. coli* TauA (shown in yellow cartoon) and *X. axonopodis* SsuA (shown in grey cartoon) structures display a high degree of structural similarity. (D) A close-up of the binding site of the *E. coli* TauA and *X. axonopodis* SsuA bound to MES show conserved interactions. Asp205 is absent in *X. axonopodis* SsuA and it is the key determinant to taurine binding. The equivalent residue in SsuA is Gly187 (shown in italic and underlined). Although Asp205 is not involved in MES coordination, it shown for clarity.

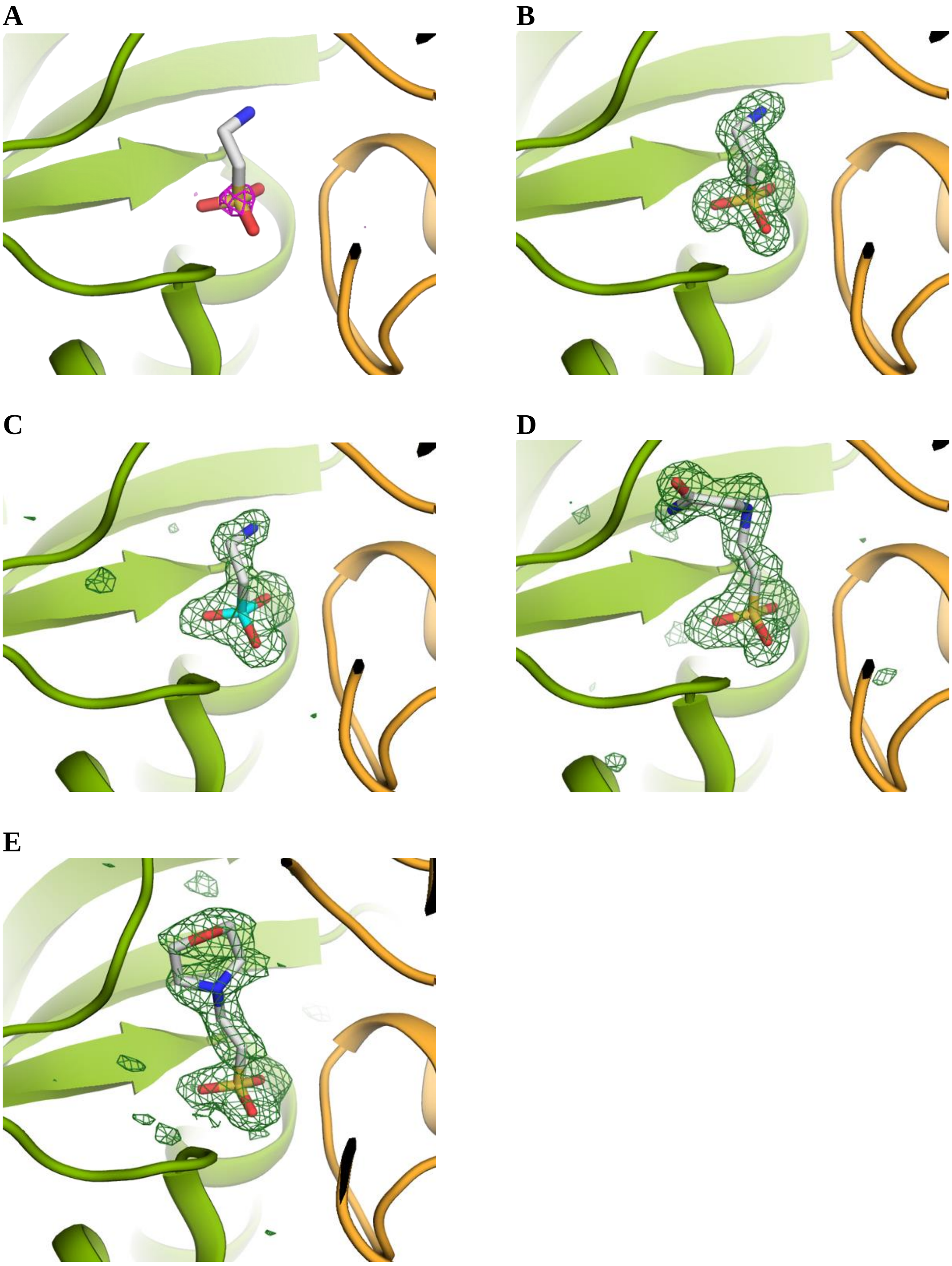

Figure S2. Electron density maps. (A) Anomalous difference map (magenta mesh) contoured at 8 σ, is shown for taurine from the long-wavelength data set, λ=2.37 Å. (B-E) Positive Fo-Fc electron density maps (green mesh) contoured at 3 σ, for (B) taurine, (C) 2-AEP, (D) ACES and (E) MES after molecular replacement. The molecules have not been included in the refinement but are shown for reference. Same colour scheme as Figure 1.

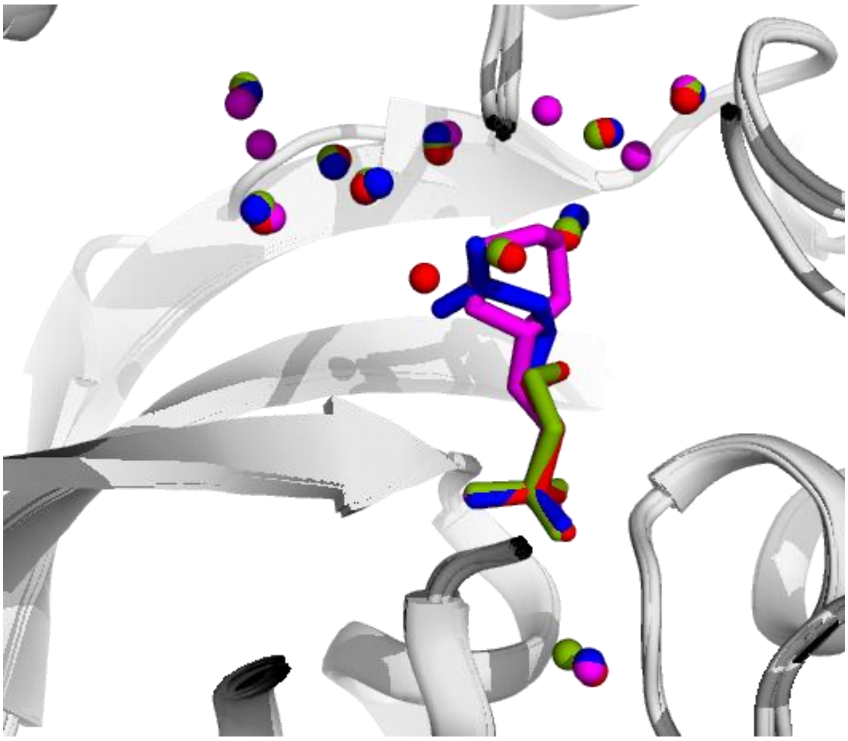

Figure S3. Water analysis from the crystal structures. All the ligand-bound TauA structures have been superimposed. There are ‘conserved’ water molecules at the proximity of the ligands, but displacement of water molecules occurs relative to the taurine structure (shown in red). Binding of ACES (shown in blue) and MES (shown in purple) results in significant water molecule displacement. Most of the waters are conserved between the taurine and 2-AEP (shown in green).
